# Cardiometabolic Response to Saroglitazar in a Young Indian Man With Obesity, Prediabetes, Insulin Resistance, and High-Normal Albuminuria: A Case Report

**DOI:** 10.7759/cureus.114699

**Published:** 2026-08-17

**Authors:** Supratik Bhattacharyya

**Affiliations:** 1 Endocrinology, Diabetes, and Metabolism, Rajarhat Apollo Sugar Clinic, Kolkata, IND

**Keywords:** albuminuria, asian indian obesity, insulin resistance, ppar-α/ppar-γ, prediabetes, saroglitazar

## Abstract

A 22-year-old Indian man presented to the outpatient clinic for a routine health check and had a body mass index (BMI) of 27.6 kg/m², which falls within the obesity range according to the Consensus Statement for Asian Indians, although it would be classified as overweight by Western cut-offs. Laboratory evaluation revealed prediabetes according to American Diabetes Association (ADA) criteria, along with elevated liver enzymes, insulin resistance, and high-normal urine albumin-to-creatinine ratio (UACR). Saroglitazar 4 mg once daily was initiated with structured lifestyle counselling. After three months of treatment, the patient demonstrated improvements in glycemic control, insulin resistance, liver enzyme levels, and urinary albumin excretion. A concurrent weight reduction of 1.3 kg was observed following initiation of saroglitazar, despite no reported changes in lifestyle modification or dietary advice compared with the two preceding visits, during which no weight change had occurred; however, this modest change may fall within ordinary biological variation, and its relative contribution from lifestyle versus pharmacological factors cannot be established from this single, uncontrolled observation. The overall direction of these changes suggests potential beneficial effects of early pharmacological intervention across metabolic, hepatic, and renal parameters. However, adequately powered controlled trials are needed before such early intervention strategies can be recommended for routine clinical use.

## Introduction

The Indian Council of Medical Research-India Diabetes (ICMR-INDIAB-17) cross-sectional survey estimates the number of Indian adults with prediabetes at ~136 million, exceeding the 101 million with diabetes [1]. The Asian Indian phenotype combines modest body mass index (BMI) with disproportionate visceral adiposity, insulin resistance, atherogenic dyslipidemia, and early microvascular involvement. Therefore, the Indian Consensus Group has long argued for ethnicity-specific BMI thresholds, defining obesity at ≥25 kg/m² rather than the ≥30 kg/m² used in Caucasian populations [2]. Misclassifying an obese Asian Indian individual as merely overweight may delay risk recognition and appropriate intervention.

Saroglitazar is a dual peroxisome proliferator-activated receptor (PPAR)-α/PPAR-γ agonist developed in India and approved for diabetic dyslipidemia and metabolic dysfunction-associated steatotic liver disease (MASLD). Its predominant PPAR-α activity lowers serum triglycerides and improves the lipid profile, while moderate PPAR-γ activity enhances peripheral insulin sensitivity [3]. The drug's role in prediabetes is less well characterized, although the Study of Saroglitazar in Treatment of Prediabetes with Dyslipidemia (STOP-D) reported favorable lipid and glycemic changes in prediabetic adults with concomitant dyslipidemia treated with saroglitazar 4 mg for 24 weeks [4].

This case report describes a young obese Indian man with prediabetes, marked insulin resistance, transaminitis, and high-normal urine albumin-to-creatinine ratio (UACR), who responded to saroglitazar combined with lifestyle modification.

Informed consent

The patient was informed that the case details would be published for scientific and educational purposes, and written informed consent was obtained for the publication of relevant clinical information.

## Case presentation

A 22-year-old Indian man presented to the outpatient clinic for a routine check-up after experiencing gradual weight gain over the preceding 18 months. He had no presenting complaints, was not on regular medication, and had no history of alcohol use, hepatotoxic exposure, premature cardiovascular disease, or liver disease. He was a non-smoker. The height was 169 cm and body weight 79.10 kg, with a BMI of 27.6 kg/m². Under the 2009 Indian Consensus thresholds (overweight: 23.0-24.9, obesity: ≥25.0 kg/m²), the patient falls within the obesity range [2]; the same value would be considered "overweight" under the Western National Institutes of Health (NIH) guidelines. Pulse was 98 beats per minute.

Laboratory findings are summarized in Table 1. Fasting plasma glucose was 105 mg/dL (reference range: 70-99 mg/dL), postprandial glucose was 173 mg/dL (reference range: <140 mg/dL), and glycosylated hemoglobin (HbA1c) was 5.8% (reference range: 4.0%-5.6%), all three indices placing him within the prediabetes range [5]. The method and timing of the postprandial glucose measurement were not further specified in the available clinical record and should not be equated with a standardized 75-g oral glucose tolerance test; accordingly, this value should not be treated as equivalent to the American Diabetes Association two-hour oral glucose tolerance test criterion. Alanine aminotransferase (ALT) (reference range: 7-40 U/L) and aspartate aminotransferase (AST) (reference range: 8-40 U/L) were 109 and 70.6 U/L, respectively (2.7× and 1.8× upper limit of normal), and alkaline phosphatase (ALP) was 121 U/L (reference range: 44-147 U/L). Homeostasis Model Assessment for Insulin Resistance (HOMA-IR) was 4.5 (reference range: <2.5) [6], well above the ~2.5 cut-off generally used in Indian cohorts to indicate clinically meaningful insulin resistance. Urine albumin-to-creatinine ratio (UACR) on a first-morning spot urine was 29.1 mg/g (reference range: <30 mg/g), below the Kidney Disease: Improving Global Outcomes (KDIGO) 30 mg/g threshold for moderately increased albuminuria but within a range now recognized to carry cardiometabolic risk on a continuous scale [7]. As this reflects a single spot measurement, the standardization of collection conditions and the availability of repeat pre-treatment measurements could not be determined from the available clinical record; urinary albumin excretion is additionally subject to biological variability related to factors such as physical activity, hydration status, and intercurrent illness, and this baseline value should therefore be interpreted with appropriate caution. Lifestyle modification with structured dietary advice had been initiated at the previous two consultations; the patient was adherent to this advice, but no appreciable change in body weight was recorded over these two visits before the initiation of saroglitazar. Therefore, saroglitazar 4 mg once daily was prescribed with lifestyle modifications owing to dysglycemia, insulin resistance, transaminitis, and high-normal UACR.

**Table 1:** Changes in glycemic, hepatic, and metabolic parameters at baseline and at three months of saroglitazar therapy ALP: alkaline phosphatase, ALT: alanine aminotransferase, AST: aspartate aminotransferase, BMI: body mass index, HOMA-IR: Homeostasis Model Assessment of Insulin Resistance, HbA1c: glycosylated hemoglobin, UACR: urine albumin-to-creatinine ratio

Parameter	Reference range	Baseline	At 3 months
Weight	-	79.10 kg	77.80 kg
BMI	18.5-22.9 kg/m² (Asian Indian cut-off)	27.6 kg/m²	27.2 kg/m²
Pulse	60-100 bpm	98 bpm	70 bpm
Fasting plasma glucose	70-99 mg/dL	105 mg/dL	89 mg/dL
Post-prandial glucose	<140 mg/dL	173 mg/dL	138 mg/dL
HbA1c (%)	4.0%-5.6%	5.8%	5.3%
ALT	7-40 U/L	109 U/L	29.4 U/L
AST	8-40 U/L	70.6 U/L	20.7 U/L
ALP	44-147 U/L	121 U/L	Not done
HOMA-IR	<2.5 (Indian cohorts)	4.5	3.3
UACR	<30 mg/g	29.1 mg/g	21.73 mg/g

The patient was reviewed in September 2025, three months after baseline (Table 1). Lifestyle modification and medical nutrition therapy were continued unchanged from the previous visits. Body weight decreased by 1.3 kg to 77.80 kg, and pulse rate was 70/min. Fasting and post-prandial glucose were 89 and 138 mg/dL, respectively, with HbA1c at 5.3%. ALT and AST were within normal limits (29.4 and 20.7 U/L). HOMA-IR was reduced to 3.3 and UACR to 21.73 mg/g. There were no reported adverse events such as gastrointestinal upset, peripheral edema, or weight gain.

## Discussion

A BMI of 27.6 kg/m² would, under the 1998 National Institutes of Health (NIH)/World Health Organization (WHO) guidelines, place the patient in the overweight category, with obesity reserved for BMI ≥ 30 kg/m². However, this is misleading to an Indian patient. South Asian adults reach a percentage body fat equivalent to a Caucasian BMI of 30 at a BMI closer to 25, and the cluster of insulin resistance and early renal risk markers associated with metabolic syndrome appears at BMI values overlapping the Caucasian "overweight" range [2]. The 2009 Indian Consensus formally set the obesity cut-off at BMI ≥ 25 kg/m² for this population. A 22-year-old with HOMA-IR of 4.5, transaminitis at almost three times the upper reference limit, and urinary albumin excretion approaching the KDIGO threshold for moderately increased albuminuria makes the case that the ethnicity-specific classification is more than semantic.

A second observation is that the four abnormalities at baseline are biologically coupled rather than incidental. Insulin resistance in obese young adults is now recognized to drive end-organ change before diabetes. Mechanistically, hyperinsulinemia favors hepatic de novo lipogenesis and intrahepatic triglyceride accumulation, and the same milieu reduces nitric oxide-mediated glomerular endothelial function and alters podocyte insulin signaling, with consequent urinary albumin leak [8]. Cardiovascular outcome data support the clinical correlate: a meta-analysis of 129 studies reported that ADA-defined prediabetes was associated with a 13% increase in composite cardiovascular events and in all-cause mortality compared with normoglycemia [9]. KDIGO treats albuminuria as a continuous variable, with high-normal UACR values still carrying prognostic weight, particularly when insulin resistance is present [7]. The combination of dysglycemia, marked insulin resistance, transaminitis, and high-normal UACR in the patient therefore represents the early metabolic-syndrome spectrum. The combination of dysglycemia, marked insulin resistance, transaminitis, and high-normal UACR suggests an early adverse cardiometabolic phenotype; however, this should not be interpreted as a formal diagnosis of metabolic syndrome; standard diagnostic parameters for metabolic syndrome (e.g., waist circumference, fasting triglycerides, high-density lipoprotein cholesterol (HDL-C), and blood pressure) were not systematically assessed in this patient.

Glycemic indices shifted from the prediabetic range toward normoglycemia during treatment, with concurrent favorable changes in HOMA-IR, transaminase levels, and UACR. In a placebo-controlled phase III PRESS-VI trial, saroglitazar 4 mg produced a 47% reduction in triglycerides over 12 weeks in patients with diabetic dyslipidemia [3], and the open-label STOP-D study demonstrated improvement in glycemic and lipid parameters in 40 prediabetic adults at 24 weeks [4]. Real-world data from Indian cohorts have shown that saroglitazar improves lipid and glycemic parameters in patients with diabetic dyslipidemia, with ALT reductions reported in the MASLD subset, consistent with PPAR-α-mediated reduction in hepatic triglyceride accumulation [10]. In the present patient, hepatic steatosis (MASLD) was not confirmed by imaging, and the observed ALT/AST normalization should not be interpreted as evidence of improvement in hepatic steatosis. The UACR change was modest in absolute terms but moved in parallel with HOMA-IR, in line with the proposed pathophysiological link between insulin resistance and glomerular permeability [8].

The case report has several limitations. This is a single, uncontrolled observation in which saroglitazar was administered concurrently with ongoing lifestyle intervention, without a pre-treatment control period, a comparator, or repeated measurements demonstrating the trajectory of each variable before drug initiation; a single observation of this kind cannot establish causality. Although structured lifestyle advice was unchanged from the two preceding visits, during which no weight change had occurred, unchanged advice does not necessarily mean unchanged dietary intake, physical activity, or adherence, and this prior stable-weight period should not be regarded as a formal control period. The 1.3 kg weight reduction is modest and may fall within ordinary biological variation; as with the other observed changes, regression to the mean (particularly relevant for the markedly abnormal baseline transaminases) cannot be separated from a pharmacological effect in this uncontrolled setting. The follow-up interval was short, and transaminase and UACR changes were captured only at a single time point without confirmation. Whether the urine albumin-to-creatinine ratio was collected under standardized conditions, and whether repeat measurements were available before treatment initiation, could not be determined from the available clinical record. The precise method and timing of the postprandial glucose measurement (e.g., whether obtained via a standardized oral glucose tolerance test or as a routine post-meal sample) were likewise not specified and could not be determined. Liver imaging and transient elastography were not obtained, so the underlying basis of the transaminitis remains undetermined, and hepatic steatosis (MASLD) was not radiologically confirmed in this patient. A complete lipid profile (triglycerides, total cholesterol, low-density lipoprotein cholesterol (LDL-C), HDL-C, and non-high-density lipoprotein cholesterol (non-HDL-C)) was not obtained at baseline or follow-up; whether this reflects a clinical decision, resource constraints, or another reason could not be determined from the available record. Given saroglitazar's predominant PPAR-α-mediated lipid activity and its principal evidence base in patients with hypertriglyceridemia or diabetic dyslipidemia, this omission limits assessment of the drug's cardiometabolic effect in this patient. Saroglitazar prescribed in prediabetes without overt dyslipidemia is off-label, and therefore, the findings should be interpreted cautiously and require confirmation in larger, controlled studies.

## Conclusions

This case showed that early initiation of saroglitazar 4 mg once daily in a young obese individual with prediabetes was associated with improvements in glycemic control, insulin resistance, and hepatic transaminases, along with a reduction in high-normal UACR. This case highlights two key priorities: the adoption of ethnicity-specific BMI cut-offs for routine risk stratification in Indian patients and the need for adequately powered controlled trials to evaluate the efficacy and safety of saroglitazar in young Indian adults with early metabolic syndrome.
